# Supplementary material for: TSniffer: unbiased de novo identification of RNA editing sites and quantification of editing activity in RNA-seq data
Source: Genome Biol. 2026 Jan 19;27:5. doi: 10.1186/s13059-026-03941-2 (PMC12838065; doi:10.1186/s13059-026-03941-2)
Supplement: Supplementary file 1 — Additional file 1: Supplementary Materials; Description: Figures S1-15, Table S1. [file 13059_2026_3941_MOESM1_ESM.pdf]

TSniffer: unbiased *de novo* identification of RNA editing sites and quantification of editing activity in RNA-seq data

Maike Herrmann[1,2], Yvonne Krebs[1,2], Francisco M. Acosta[3,4,5], Sebastian Parusel[3], Oliver Siering[1,3], Felix G. M. Andres[1], Biruhalem Taye[3], Csaba Miskey[6], and Christian K. Pfaller[1,3,4,7*]

[1] Division of Veterinary Medicine, Paul-Ehrlich-Institute, Langen, Germany
[2] Host-Pathogen Interactions, Paul-Ehrlich-Institute, Langen, Germany
[3] Department of Molecular Medicine, Mayo Clinic, Rochester, MN, United States
[4] Virology and Gene Therapy Track, Mayo Clinic Graduate School of Biomedical Sciences, Rochester, MN, United States
[5] X: @Matt_a_photo
[6] Division of Hematology, Cell and Gene Therapy, Paul-Ehrlich-Institute, Langen, Germany
[7] X: @ckpfaller

Supplementary Materials

This file contains supplementary **Fig. S1-S15** and supplementary **Table S1**.

Supplementary Figures

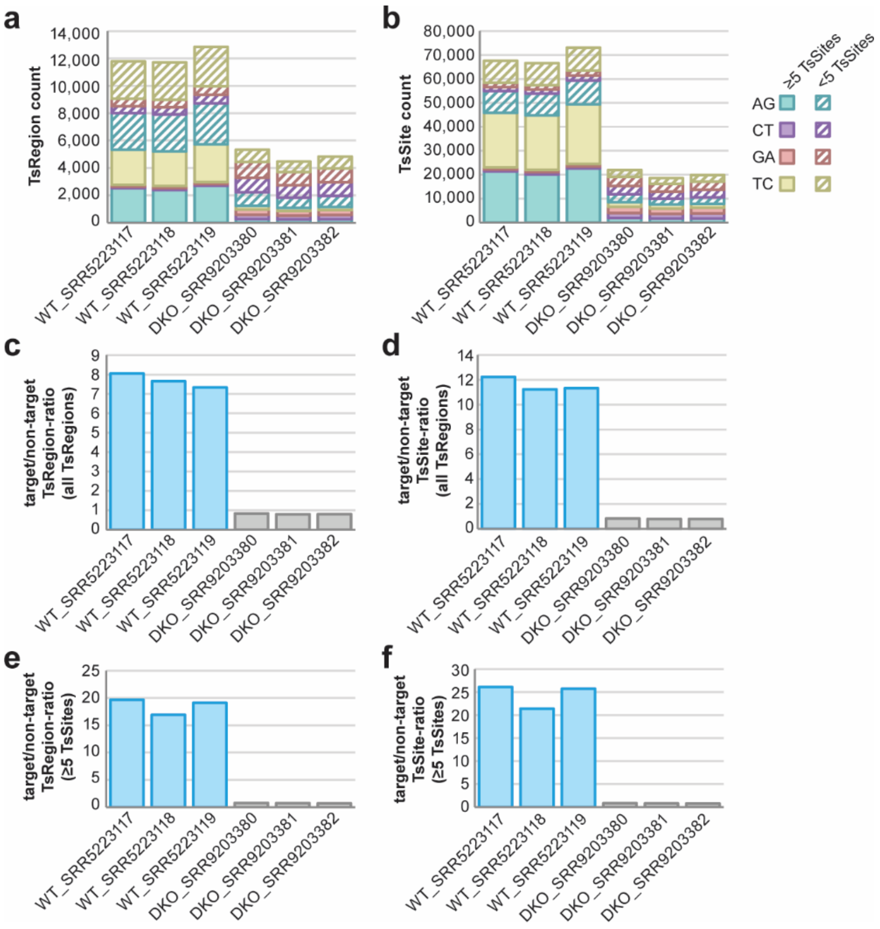

Fig. S1: *TSniffer de novo* analysis of individual WT and DKO mouse brain datasets. (**a**) TsRegion count and (**b**) TsSite count of *de novo*-identified TsRegions in three individual BAM alignments of wild type (WT) and ADAR1/2-deficient (DKO) mouse brains. (**c**) Ratio of target (AG + TC) to non-target (CT + GA) TsRegion counts in each dataset. (**d**) Ratio of target (AG + TC) to non-target (CT + GA) TsSite counts in each dataset. (**e**) Ratio of target (AG + TC) to non-target (CT + GA) TsRegion counts in each dataset. Only TsRegions with at least 5 TsSites are included in this calculation. (**f**) Ratio of target (AG + TC) to non-target (CT + GA) TsSite counts in each dataset. Only TsSites from TsRegions with at least 5 TsSites are included in this calculation.

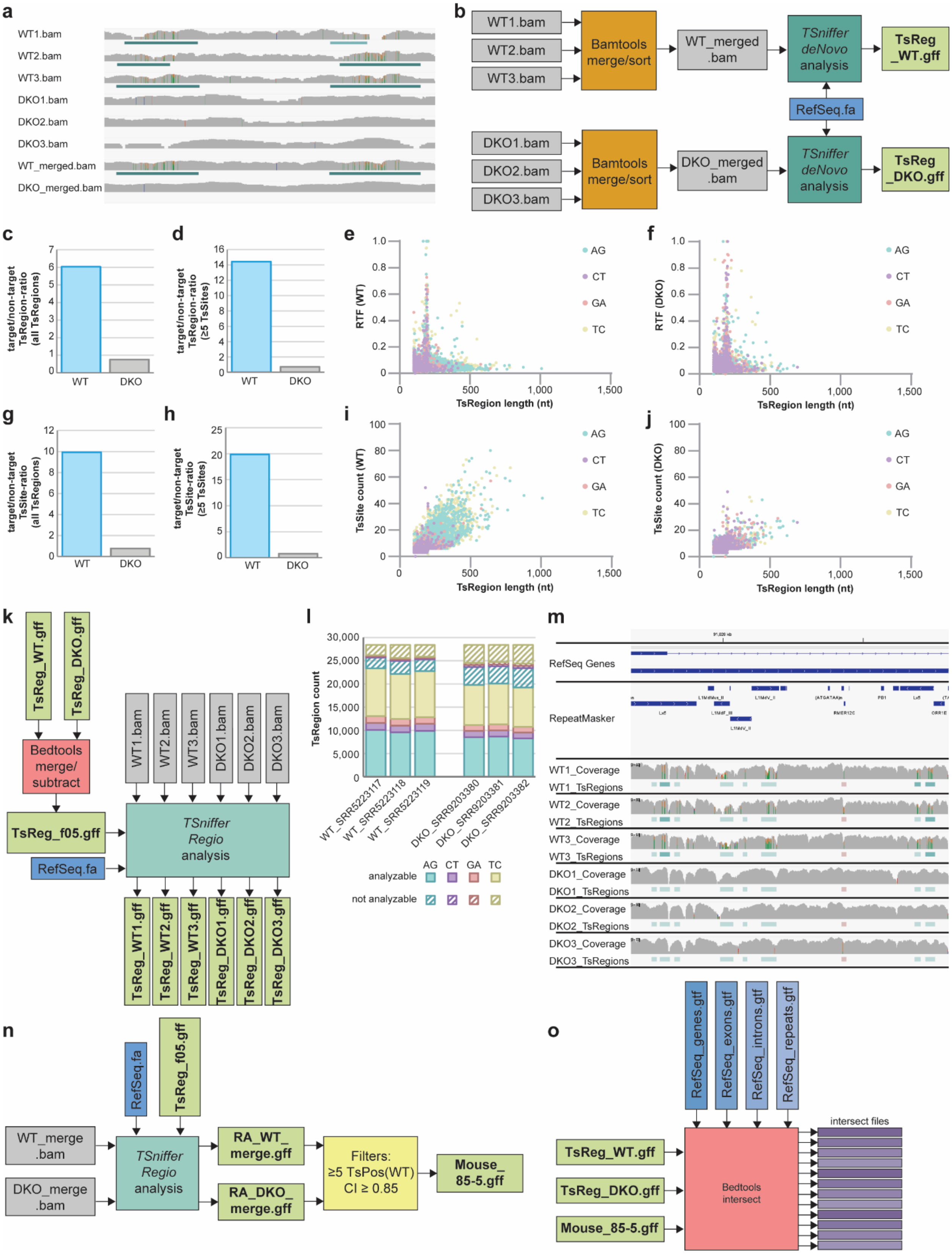

Fig. S2: Optimized *TSniffer* pipeline and downstream analysis. (**a**) BAM alignment coverage plots of a genomic region exhibiting low coverage gaps at sites of RNA editing in individual datasets (n=3 for WT and DKO). Gaps can be restored through the merging of the individual datasets. (**b**) Workflow of *TSniffer deNovo* analysis on combined BAM alignments from biological replicates. (**c**) Ratio of target (AG + TC) to non-target (CT + GA) TsRegion counts in combined BAM datasets. (**d**) Ratio of target (AG + TC) to non-target (CT + GA) TsRegion counts in

combined BAM datasets. Only TsRegions with at least 5 TsSites are included in this calculation. (**e**) Distribution of TsRegion length and RTF values of TsRegions in the combined WT dataset. Different TsTypes are color-coded. (**f**) Distribution of TsRegion length and RTF values of TsRegions in the combined DKO dataset. (**g**) Ratio of target (AG + TC) to non-target (CT + GA) TsSite counts in combined BAM datasets. (**h**) Ratio of target (AG + TC) to non-target (CT + GA) TsSite counts in combined BAM datasets. Only TsSites of TsRegions with at least 5 TsSites are included in this calculation. (**i**) Distribution of TsRegion length and TsSite counts of TsRegions in the combined WT dataset. Different TsTypes are color-coded. (**j**) Distribution of TsRegion length and TsSite counts of TsRegions in the combined DKO dataset. (**k**) Workflow for removal of TsRegions shared between WT and DKO samples (TsReg_f05) and downstream *TSniffer Regio* analysis of individual BAM alignments with this set of TsRegions. (**l**) TsRegion count of *TSniffer Regio* analysis of individual BAM alignments with the TsReg_f05 set. Hashed bars indicate proportion of TsRegions returning no analyzable value due to low coverage in the alignment. (**m**) Screenshot of an alignment of a transcript region in IGV. Coverage plots (allele frequency threshold set to 0.05) and *TSniffer Regio* analysis GFF files were loaded. Darker colors of TsRegions indicate higher RTF values. (**n**) Workflow for establishing a highly representative and specific subset of TsRegions (Mouse_85-5). For details see methods. (**o**) Workflow for downstream BEDTOOLS analysis of identified TsRegions.

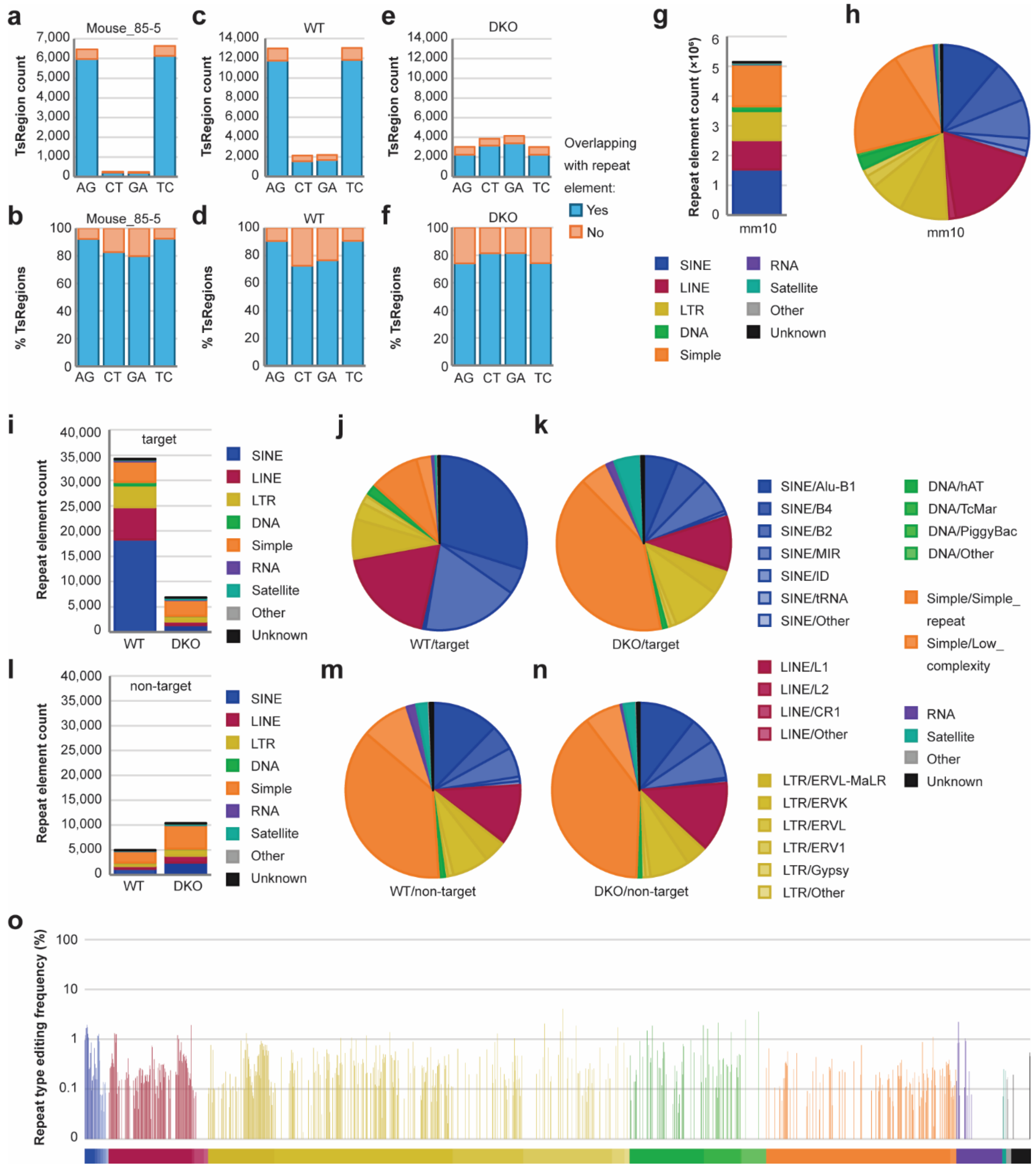

Fig. S3: ADAR editing in murine repeat elements. (**a**) Mouse_85-5 TsRegion count on intersection with annotated repeat elements. (**b**) Relative frequencies of Mouse_85-5 TsRegions overlapping with repeat elements. (**c**) Mouse WT *de novo* TsRegion count on intersection with annotated repeat elements. (**d**) Relative frequencies of Mouse WT *de novo* TsRegions overlapping with repeat elements. (**e**) Mouse DKO *de novo* TsRegion count on intersection with annotated repeat elements. (**f**) Relative frequencies of Mouse DKO *de novo* TsRegions overlapping with repeat elements. (**g**) Total repeat element count and (**h**) relative occurrence of repeat element subtypes in the mouse reference genome mm10. (**i**) Total counts of repeat element families overlapping with target TsRegions (AG + TC) in WT and DKO *de novo* datasets. (**j**) Relative frequencies of mouse repeat subtypes overlapping with target TsRegions in the WT *de novo* dataset. (**k**) Relative frequencies of mouse repeat subtypes overlapping with target TsRegions in the DKO *de novo* dataset. (**l**) Total counts of repeat element families overlapping with non-target TsRegions (CT + GA) in WT and DKO *de novo* datasets. (**m**) Relative frequencies of mouse repeat subtypes

overlapping with non-target TsRegions in the WT *de novo* dataset. (**n**) Relative frequencies of mouse repeat subtypes overlapping with non-target TsRegions in the DKO *de novo* dataset. (**o**) Frequencies of TsRegion-harboring repeat elements by specific repeat type. Color coding as in **i** – **n**.

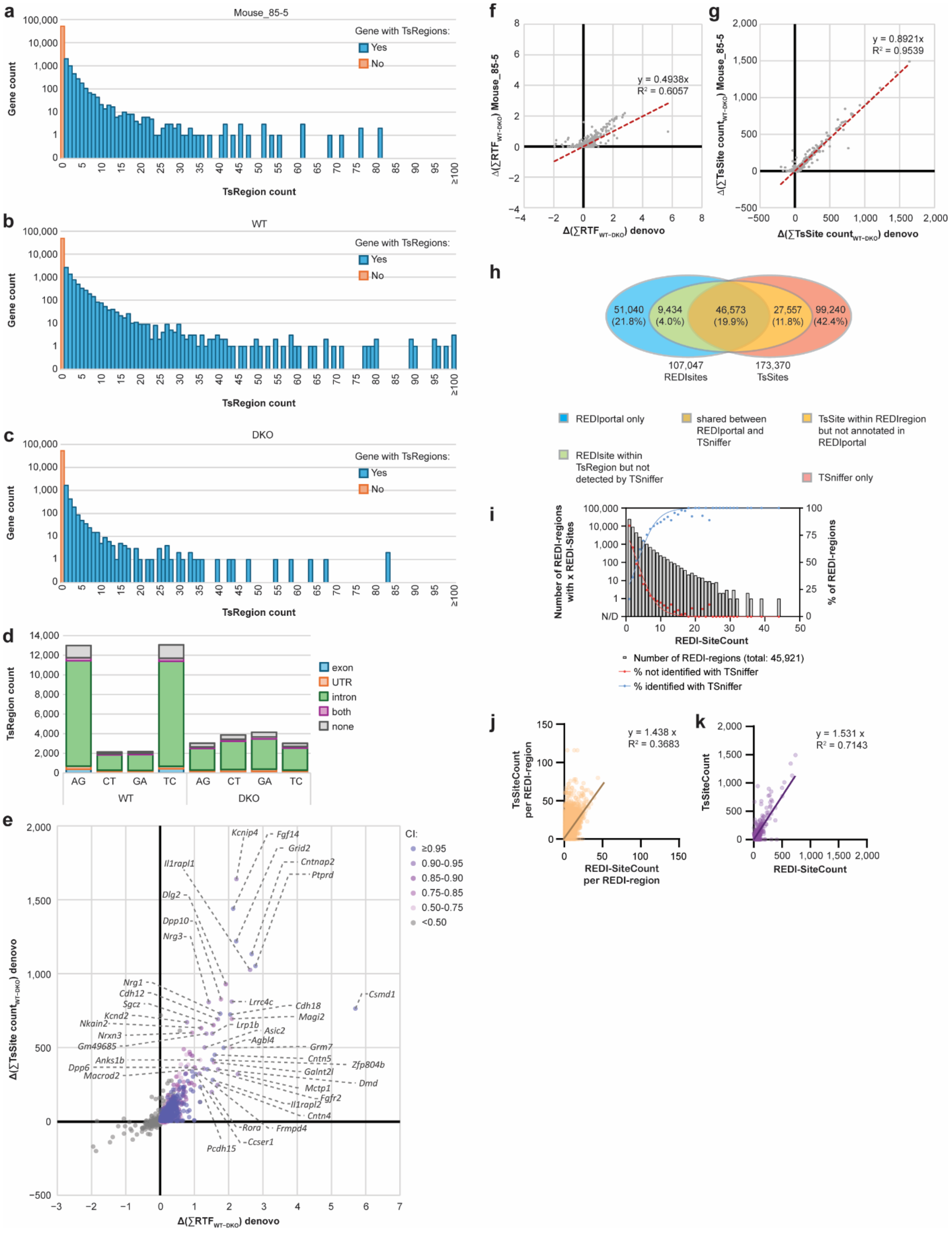

Fig. S4: Consistency between *TSniffer de novo* and Mouse_85-5 subset analyses. (**a**) Counts of genes harboring specific numbers of TsRegions of the Mouse_85-5 set. (**b**) Counts of genes harboring specific numbers of TsRegions of the Mouse WT *de novo* set. (**c**) Counts of genes harboring specific numbers of TsRegions of the Mouse DKO *de novo* set. (**d**) Distribution of WT and DKO *de novo* TsRegions across transcript elements. (**e**) Quantification of ADAR editing in mouse transcripts. Relative transition frequency (RTF) values and TsSite counts

of all Mouse WT *de novo* TsRegions intersecting with individual genes were summed up and values of respective TsRegions in DKO were subtracted. Transcripts are color-coded by the total number of calculated TsSites. Most highly edited transcripts are indicated. (**f**) Correlation of gene-specific RTF values derived from the Mouse_85-5 set and from the subtractive *de novo* analysis. (**g**) Correlation of gene-specific TsSite counts derived from the Mouse_85-5 set and from the subtractive *de novo* analysis. (**h**) Venn diagram showing overlap of TsSites included in the Mouse_85-5 dataset with editing sites annotated in the REDIportal database (REDIsites) (49). (**i**) Performance of *TSniffer* to detect REDIsites in dependence of hyperediting cluster size. (**j**) Correlation of TsSites and annotated REDIsites within hyperediting clusters. (**k**) Correlation of TsSites and annotated REDIsites per gene.

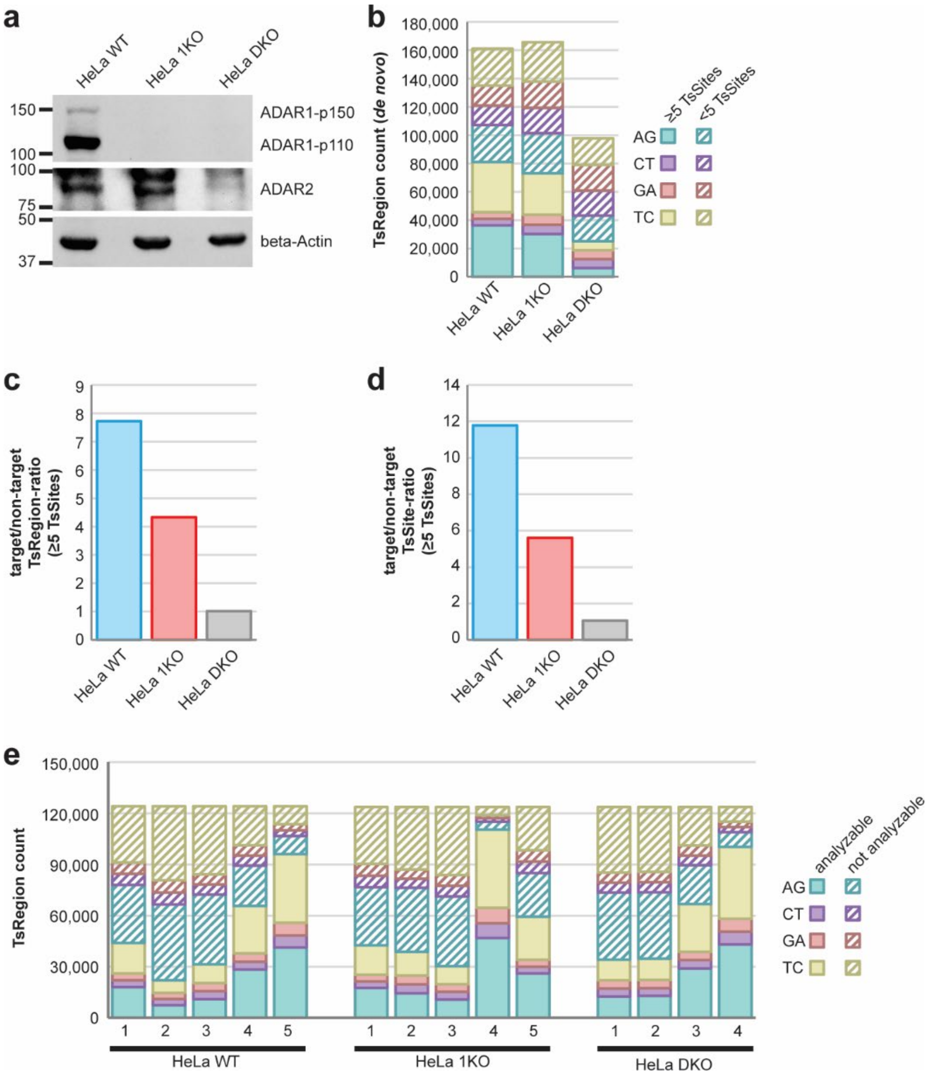

Fig. S5: Generation of ADAR1- and ADAR1/2-deficient HeLa cell lines and initial *TSniffer* analysis. (**a**) Western blot analysis of wild type (WT), ADAR1 knockout (1KO) and ADAR1/2 knockout (DKO) HeLa cells. Uncropped images are presented in **Fig. S15**. (**b**) TsRegion count of *TSniffer de novo* analyses of combined datasets of BAM alignments of biological replicates of HeLa WT (n=5), HeLa 1KO (n=5), and HeLa DKO (n=4). (**c**) Ratio of target (AG + TC) to non-target (CT + GA) TsRegion counts in combined BAM datasets. Only TsRegions with at least 5 TsSites are included in this calculation. (**d**) Ratio of target (AG + TC) to non-target (CT + GA) TsSite counts in combined BAM datasets. Only TsSites of TsRegions with at least 5 TsSites are included in this calculation. (**e**) *TSniffer* region analysis of individual BAM alignments derived from HeLa WT (n=5), HeLa 1KO (n=5), and HeLa DKO (n=4) using the HeLa WT-specific set of TsRegions. Hashed bars indicate TsRegions returning non-analyzable values in each dataset due to low coverage.

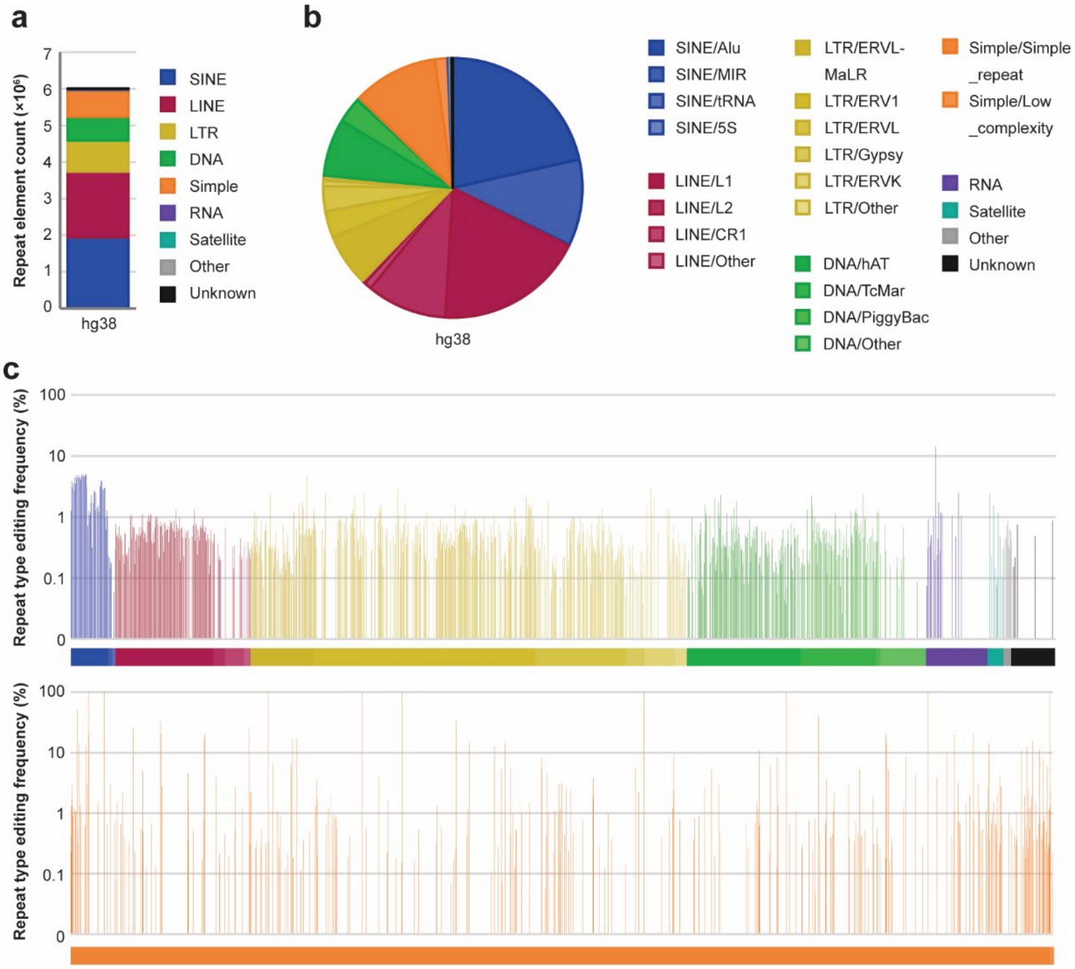

Fig. S6: Human repeat elements and frequency of ADAR editing within these elements. (**a**) Total repeat element count and (**b**) relative occurrence of repeat element subtypes in the human reference genome hg38. (**c**) Frequencies of TsRegion-harboring repeat elements by specific repeat type. Color coding as in **a** – **b**. Due to the high number of annotated simple repeats in hg38, these are shown in a separate diagram (bottom).

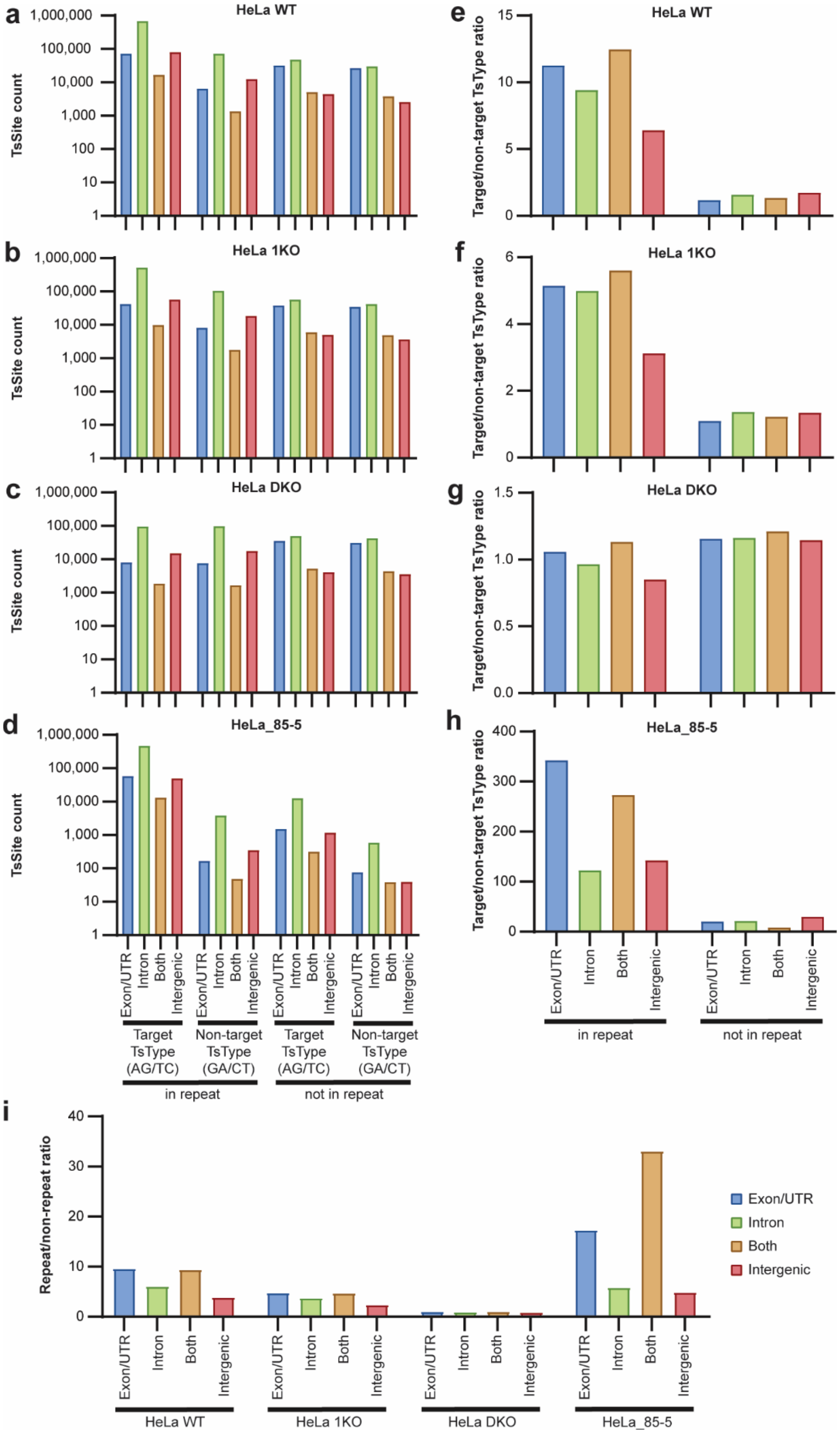
a
HeLa WT
TsSite count
1,000,000
100,000
10,000
1,000
100
10
1
b
HeLa 1KO
c
HeLa DKO
d
HeLa_85-5
Exon/UTR
Intron
Both
Intergenic
Target TsType (AG/TC)
Non-target TsType (GA/CT)
in repeat
not in repeat
e
f
g
h
Target/non-target TsType ratio
15
10
5
0
6
4
2
1.5
1.0
0.5
0.0
400
300
200
100
i
Repeat/non-repeat ratio
40
30
20
HeLa WT
HeLa 1KO
HeLa DKO
HeLa_85-5

Fig. S7: Detection of target and non-target TsSites in different genetic elements. Data is stratified by genetic elements (exon/UTR, intron, both, intergenic), TsType (target = AG, TC; non-target = CT, GA), and presence of repeat element. (**a**) Total number of TsSites detected by *TSniffer deNovo* in HeLa WT cells. (**b**) Total number of TsSites detected by *TSniffer deNovo* in HeLa 1KO cells. (**c**) Total number of TsSites detected by *TSniffer deNovo* in HeLa DKO cells. (**d**) Total number of TsSites included in the HeLa_85-5 dataset. (**e**) Ratio of target to non-target TsTypes in HeLa WT cells. (**f**) Ratio of target to non-target TsTypes in HeLa 1KO cells. (**g**) Ratio of target to non-target TsTypes in HeLa DKO cells. (**h**) Ratio of target to non-target TsTypes in the HeLa_85-5 dataset. (i) Ratio of TsSites in repeats over not in repeats for the different datasets.

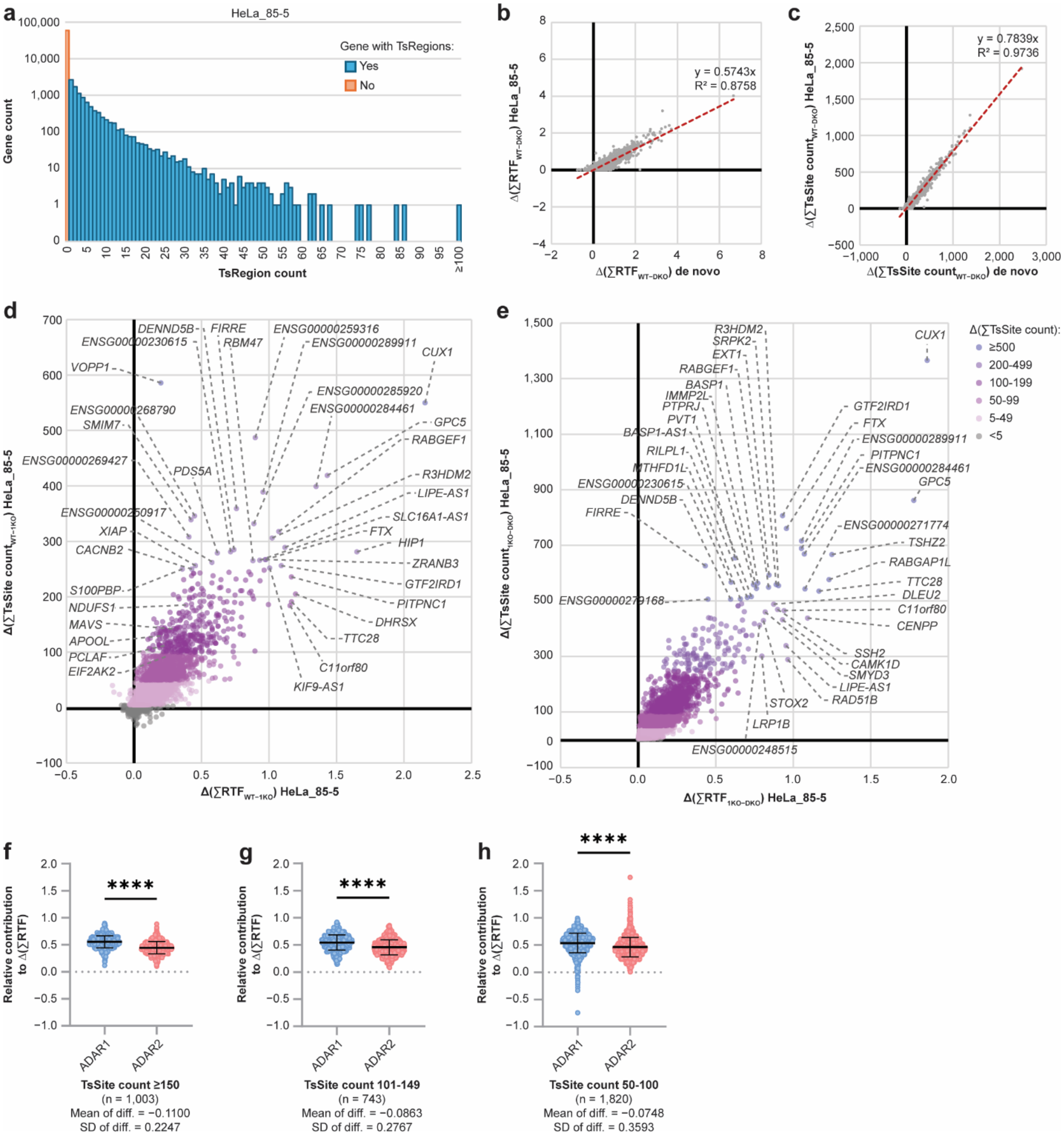

Fig. S8: Differential editing of ADAR1 and ADAR2 in human transcripts. (**a**) Counts of genes harboring specific numbers of TsRegions of the HeLa_85-5 set. (**b**) Correlation of gene-specific RTF values derived from the Mouse_85-5 set and from the subtractive *de novo* analysis. (**c**) Correlation of gene-specific TsSite counts derived from the Mouse_85-5 set and from the subtractive *de novo* analysis. (**d**) Quantification of the ADAR1-specific portion of editing in human transcripts. Relative transition frequency (RTF) values and TsSite counts of all HeLa_85-5 TsRegions intersecting with individual genes in WT were summed up and residual values of respective regions in 1KO were subtracted. Transcripts are color-coded by the total number of calculated TsSites. Most highly edited transcripts are indicated. (**e**) Quantification of the ADAR2-specific portion of editing in human transcripts. Relative transition frequency (RTF) values and TsSite counts of all HeLa_85-5 TsRegions intersecting with individual genes in 1KO were summed up and residual values of respective regions in DKO were subtracted. Transcripts are color-coded by the total number of calculated TsSites. Most highly edited transcripts are indicated. (**f**) Comparison of contribution of ADAR1 and ADAR2 to RTF values of genes with at least 150 TsSites (n = 1,003). (**g**) Comparison of contribution of ADAR1 and ADAR2 to RTF values of genes with 101-149 TsSites (n =

743). (**h**) Comparison of contribution of ADAR1 and ADAR2 to RTF values of genes with 50-100 TsSites. In **f** – **h**, paired two-tailed Student's t-test was performed between groups to test for significant differences. **** P<0.0001.

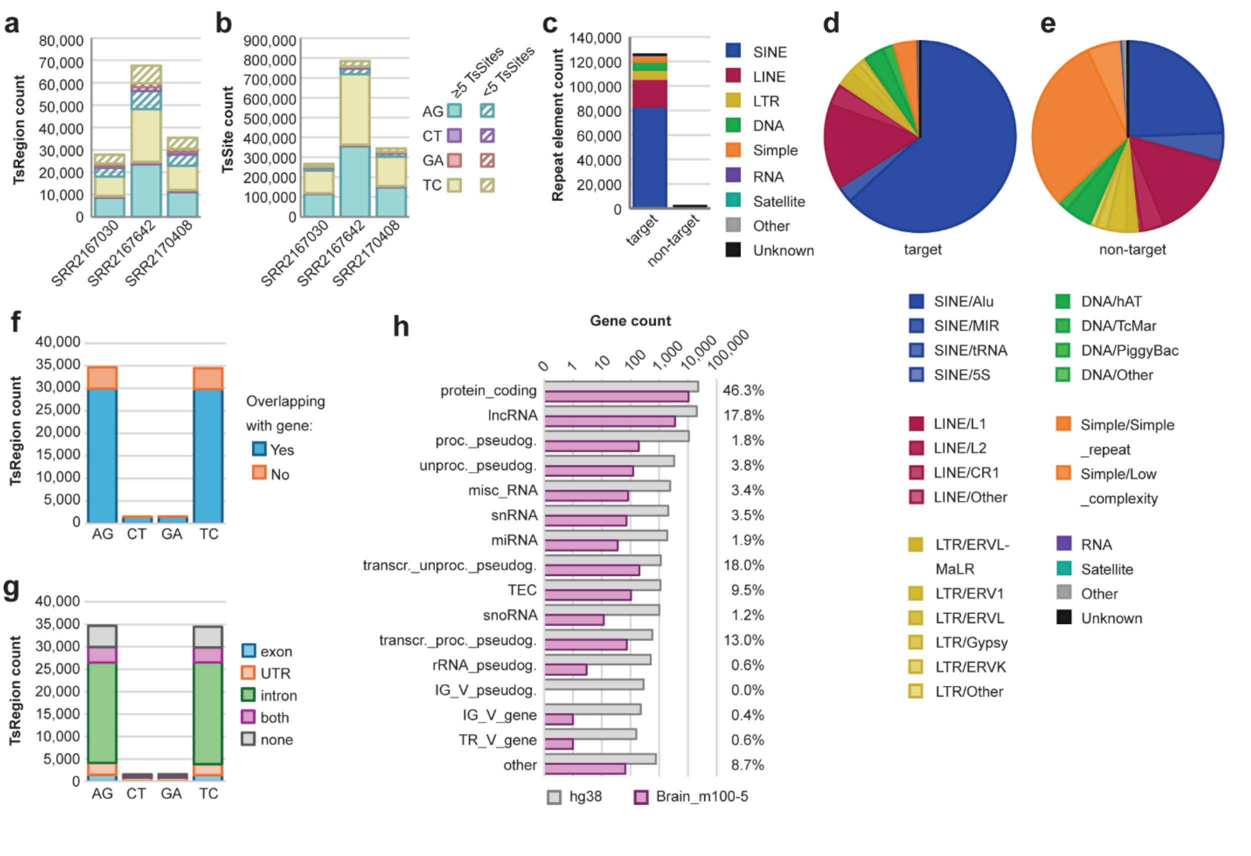

Fig. S9: ADAR editing in GTEx datasets of primary human brain samples. (**a**) TsRegion counts and (**b**) TsSite counts of *TSniffer deNovo* analyses of three GTEx brain samples. (**c**) Number of repeat elements overlapping with TsRegions of the Brain_m100-5 set. Target TsRegions are AG and TC, non-target TsRegions are CT and GA. Colors indicate different repeat families. (**d**) Relative abundance of repeat family subtypes among target TsRegion-containing repeats. (**e**) Relative abundance of repeat family subtypes among non-target TsRegion-containing repeats. (**f**) TsRegion count by intersection with annotated genes. (**g**) Distribution of TsRegions within transcript elements. (**h**) Total number of annotated genes in the hg38 reference genome (grey), total number of genes with target TsRegions (pink), and relative frequency of TsRegion-harboring genes, per gene type.

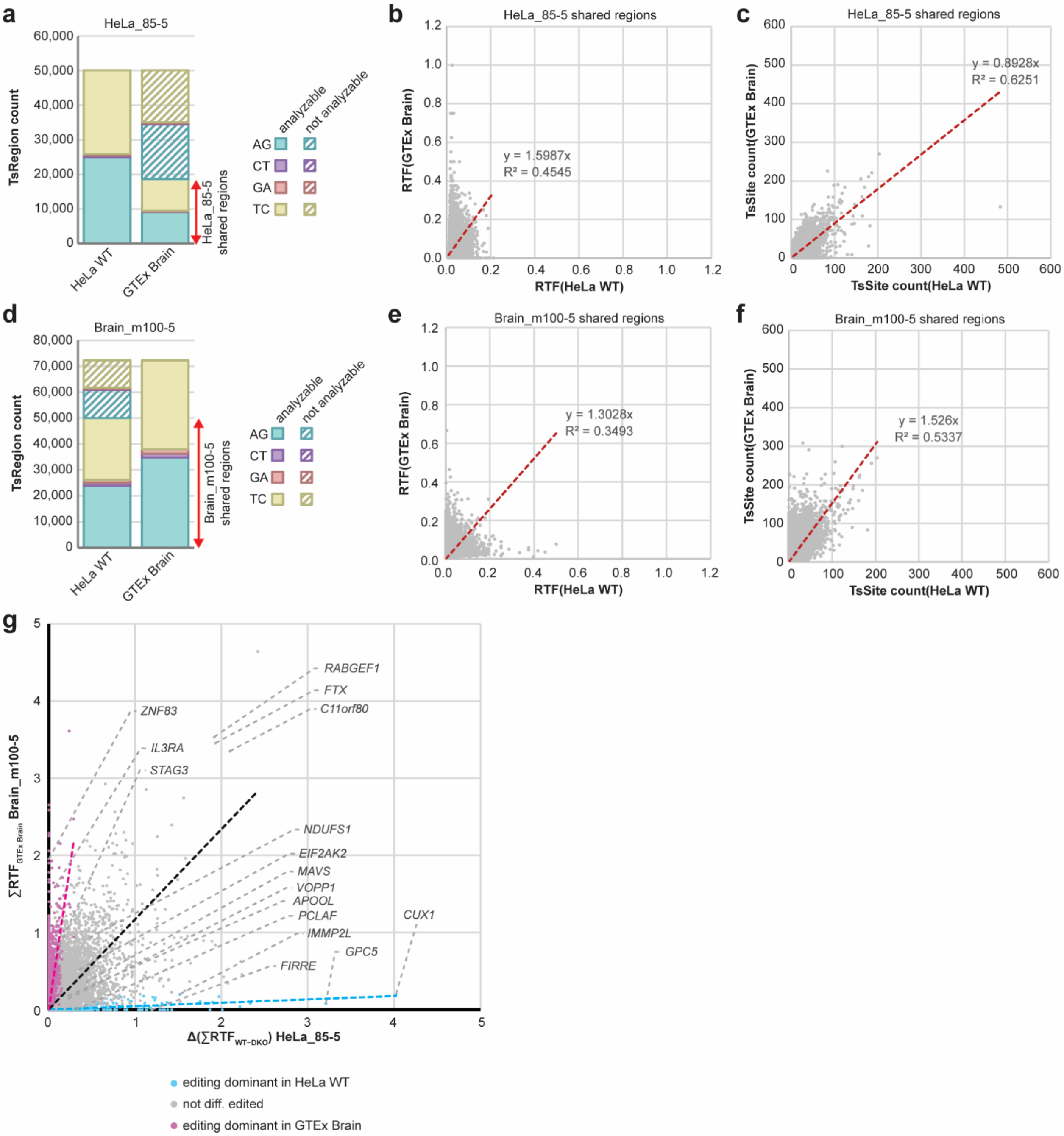

Fig. S10: Comparison of ADAR editing in HeLa cells and primary human brain tissue. (**a**) TsRegion count of *TSniffer Regio* analysis of HeLa WT and GTEx Brain datasets using the HeLa_85-5 set of TsRegions. Hashed bars indicate TsRegions returning not analyzable values in the respective dataset due to low coverage. Red arrow indicates the subset of TsRegions used for correlation analysis in **b** and **c**. (**b**) Correlation of RTF values obtained from HeLa WT cells (x-axis) and the GTEx Brain dataset (y-axis) for HeLa_85-5 TsRegions that were expressed in both datasets. (**c**) Correlation of TsSite counts obtained from HeLa WT cells (x-axis) and the GTEx Brain dataset (y-axis) for HeLa_85-5 TsRegions that were expressed in both datasets. (**d**) TsRegion count of *TSniffer Regio* analysis of HeLa WT and GTEx Brain datasets using the Brain_m100-5 set of TsRegions. Hashed bars indicate TsRegions returning not analyzable values in the respective dataset due to low coverage. Red arrow indicates the subset of TsRegions used for correlation analysis in **e** and **f**. (**e**) Correlation of RTF values obtained from HeLa WT cells (x-axis) and the GTEx Brain dataset (y-axis) for Brain_m100-5 TsRegions that were expressed in both datasets. (**f**) Correlation of TsSite counts obtained from HeLa WT cells (x-axis) and the GTEx Brain dataset (y-axis) for Brain_m100-5 TsRegions that were expressed in both datasets. (**g**) Correlation of gene-associated total RTF values

detected in HeLa WT cells using the HeLa_85-5 set (x-axis) with values from human brain samples using the Brain_m100-5 set (y-axis). Color coding if gene was dominantly edited in HeLa WT (blue; TsSite count[HeLa WT] ≥ 9 × TsSite count[Brain]) or if gene was dominantly edited in brain (pink; TsSite count[Brain] ≥ 9 × TsSite count[HeLa WT]. Dashed lines indicate linear regression for the different groups.

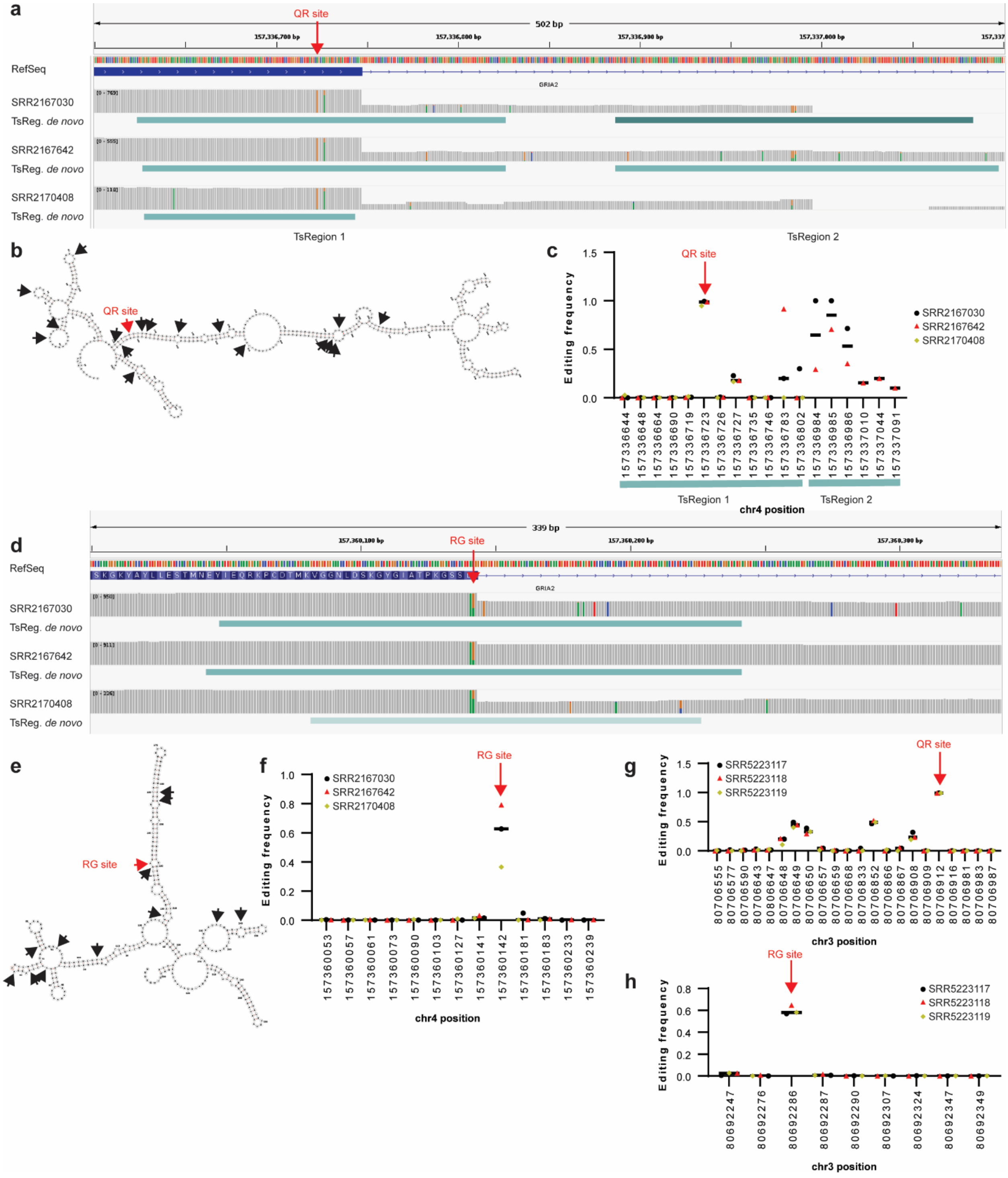

Fig. S11: Detection of site-specific editing of the *GRIA2* transcript in human and mouse brain. (**a**) Coverage plots of the *GRIA2* Q/R-site in the three GTEx human brain samples and detected TsRegions. (**b**) Secondary structure prediction of the sequence shown in **a**. Arrows indicate TsSites detected by *TSniffer deNovo*. (**c**) Editing frequencies at TsSites. (**d**) Coverage plots of the *GRIA2* R/G-site in the three GTEx human brain samples and detected TsRegions. (**e**) Secondary structure prediction of the sequence shown in **d**. Arrows indicate TsSites detected by *TSniffer deNovo*. (**f**) Editing frequencies at TsSites. (**g**) Editing frequencies at TsSites of the mouse *Gria2* Q/R-site. (h) Editing frequencies at TsSites of the mouse *Gria2* R/G-site.

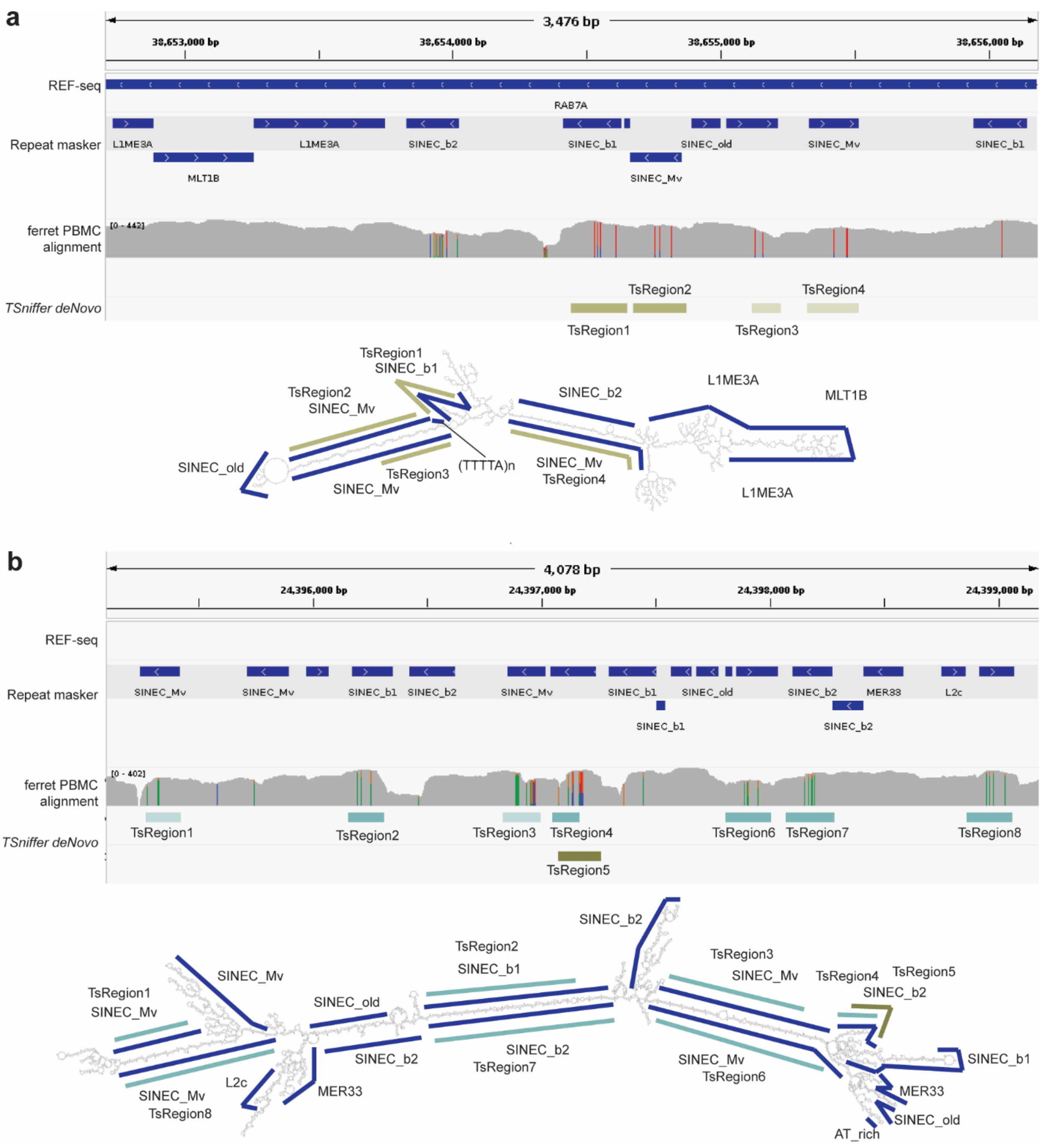

Fig. S12: Structural basis for editing of ferret repeat elements. (**a**) Coverage plots of BAM alignment and predicted secondary structure of a partial sequence of the *RAB7A* gene. Repeat elements identified by Repeat masker are shown in blue, identified TsRegions in gold. (**b**) Coverage plots of BAM alignment and predicted secondary structure of a partial sequence downstream of the *ECE1* gene. Repeat elements identified by Repeat masker are shown in blue, identified TsRegions in teal (AG) and gold (TC).

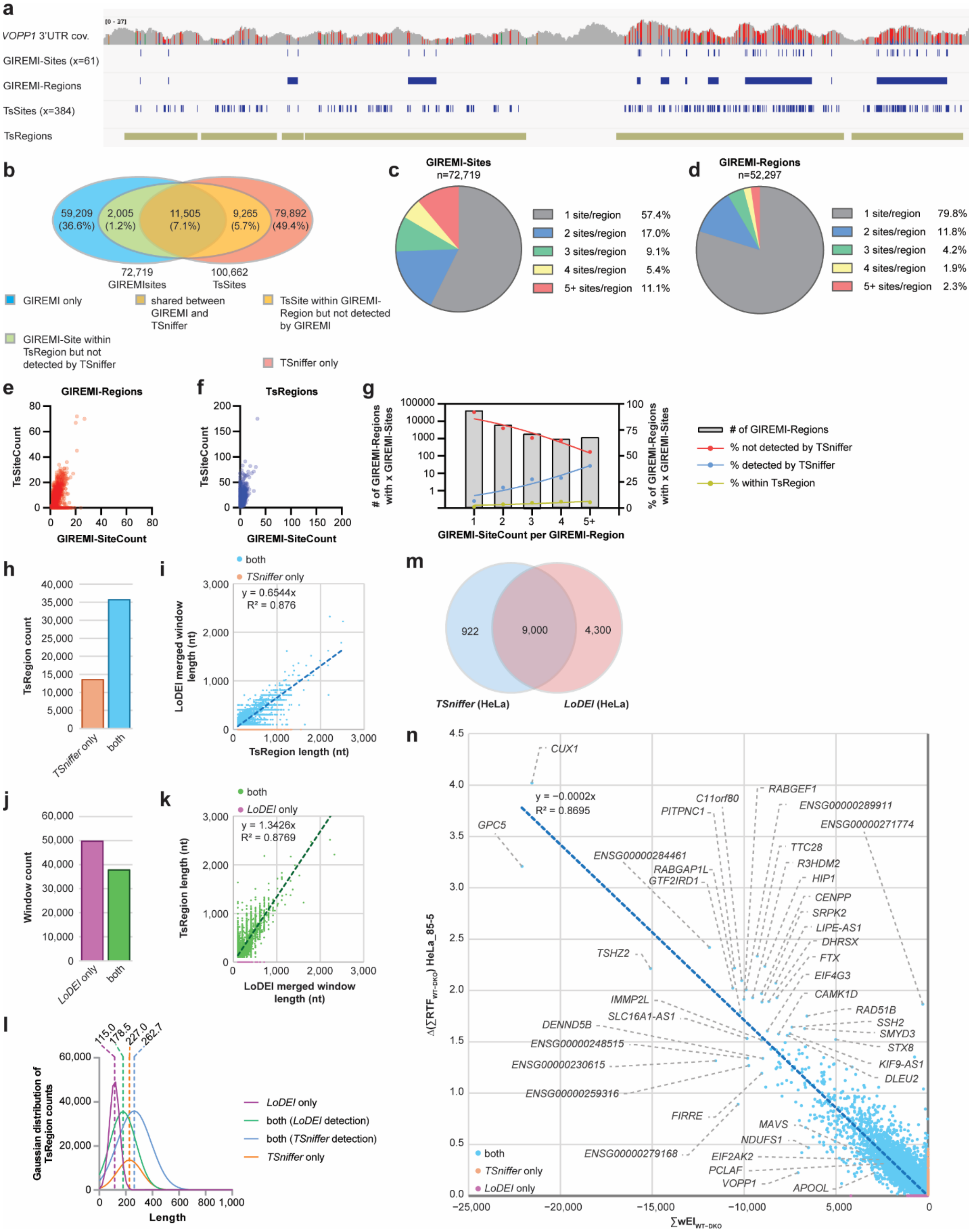

Fig. S13: Performance comparison of *TSniffer* with other approaches to detect and quantify RNA editing. (**a**) Coverage plot of the *VOPP1* 3'UTR in HeLa WT cells (dataset #1). Editing sites detected by GIREMI (GIREMI-Sites) or *TSniffer deNovo* (TsSites) are indicated in blue. GIREMI-Regions are not a direct output from GIREMI but were defined as regions where neighboring editing sites occur within 100 nt distance. TsRegions indicated in gold. (**b**) Venn diagram showing overlap of TsSites and GIREMI-Sites in the HeLa WT #1 sample. (**c**) Cluster analysis of GIREMI-Sites. Percentage of GIREMI-Sites within regions containing x sites. (**d**) GIREMI-Region analysis. Percentage of GIREMI-Regions with x GIREMI-sites. (**e**) Correlation of TsSite counts and GIREMI-Site counts in

GIREMI-Regions. (**f**) Correlation of TsSite counts and GIREMI-Site counts in TsRegions. (**g**) Performance of *TSniffer* to detect GIREMI-Sites in dependence of hyperediting cluster size. (**h**) Number of HeLa_85-5 TsRegions detected by *TSniffer* only (orange) or by *TSniffer* and LoDEI (blue). (**i**) Comparison of the TsRegion length calculated by *TSniffer* and the cumulative length of overlapping significant windows detected by LoDEI. (**j**) Number of merged adjacent windows detected by LoDEI only (purple) or by LoDEI and *TSniffer* (green). (**k**) Comparison of the cumulative length of windows detected by LoDEI and overlapping TsRegions calculated by *TSniffer*. (**l**) Gaussian distribution of window/TsRegion length in all four groups (**h** – **k**). Dashed lines and values on top indicate the mean region length for each group. (**m**) Number of gene transcripts in HeLa cells identified by *TSniffer* (HeLa_85-5 set), or LoDEI, and overlap between the two analyses. (**n**) Correlation of the cumulative window editing index (wEI) for each transcript as calculated by LoDEI between HeLa WT and DKO samples, and the cumulative RTF values as calculated by *TSniffer* for the same datasets.

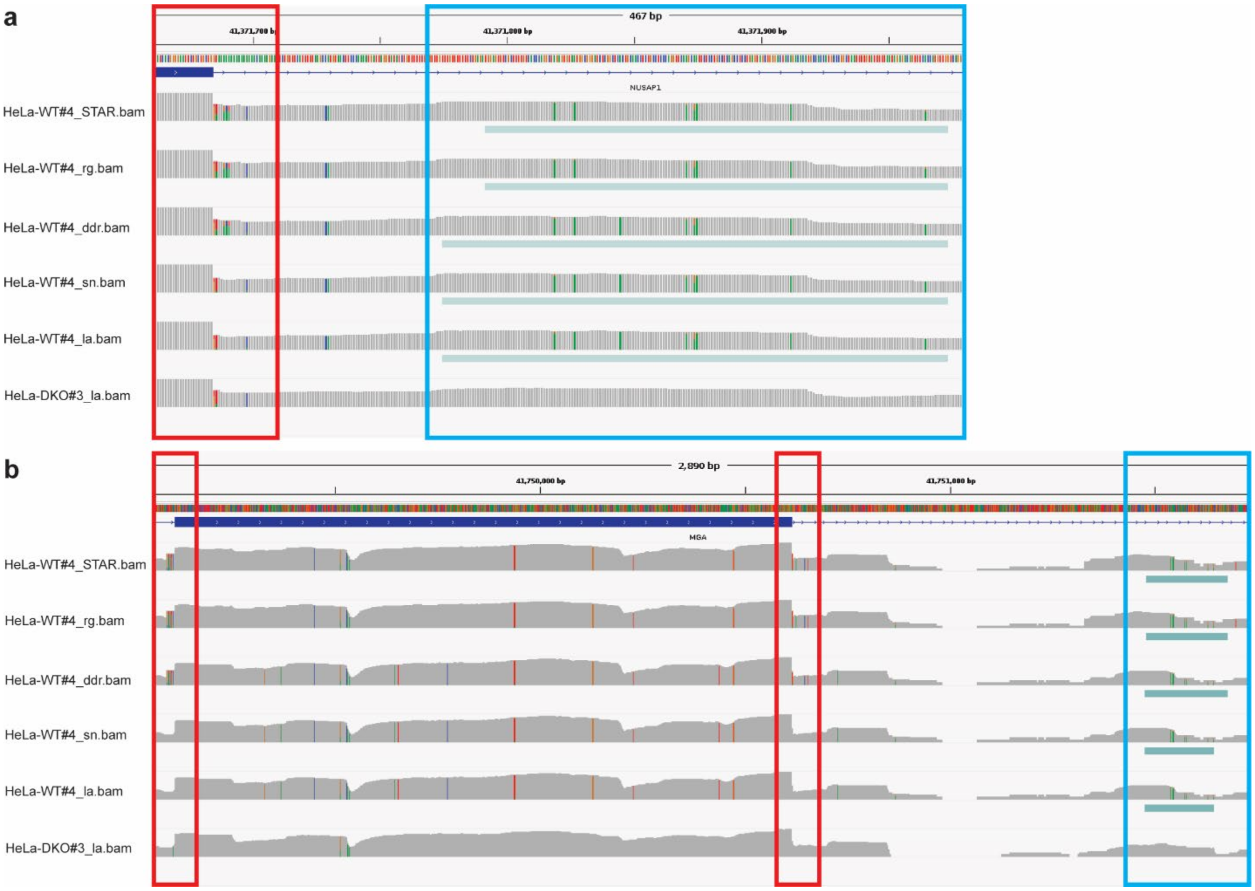

Fig. S14: Alignment errors at exon/intron junctions are ignored by *TSniffer*. BAM alignment coverage plots from the HeLa WT #4 sample are shown at different stages of RNA-seq pipeline. STAR: Output of STAR aligner. rg: BAM output after GATK AddOrReplaceReadGroups. ddr: BAM output after PICARD MarkDuplicates. sn: BAM output after GATK SplitNCigarReads. la: final BAM output after GATK LeftAlignIndels. Red boxes indicate exon/intron junctions. Light blue boxes indicate TsRegions detected by *TSniffer deNovo*. (**a**) *NUSAP1* exon 8-intron-junction. (**b**) *MGA* intron-exon 17-intron-junction.

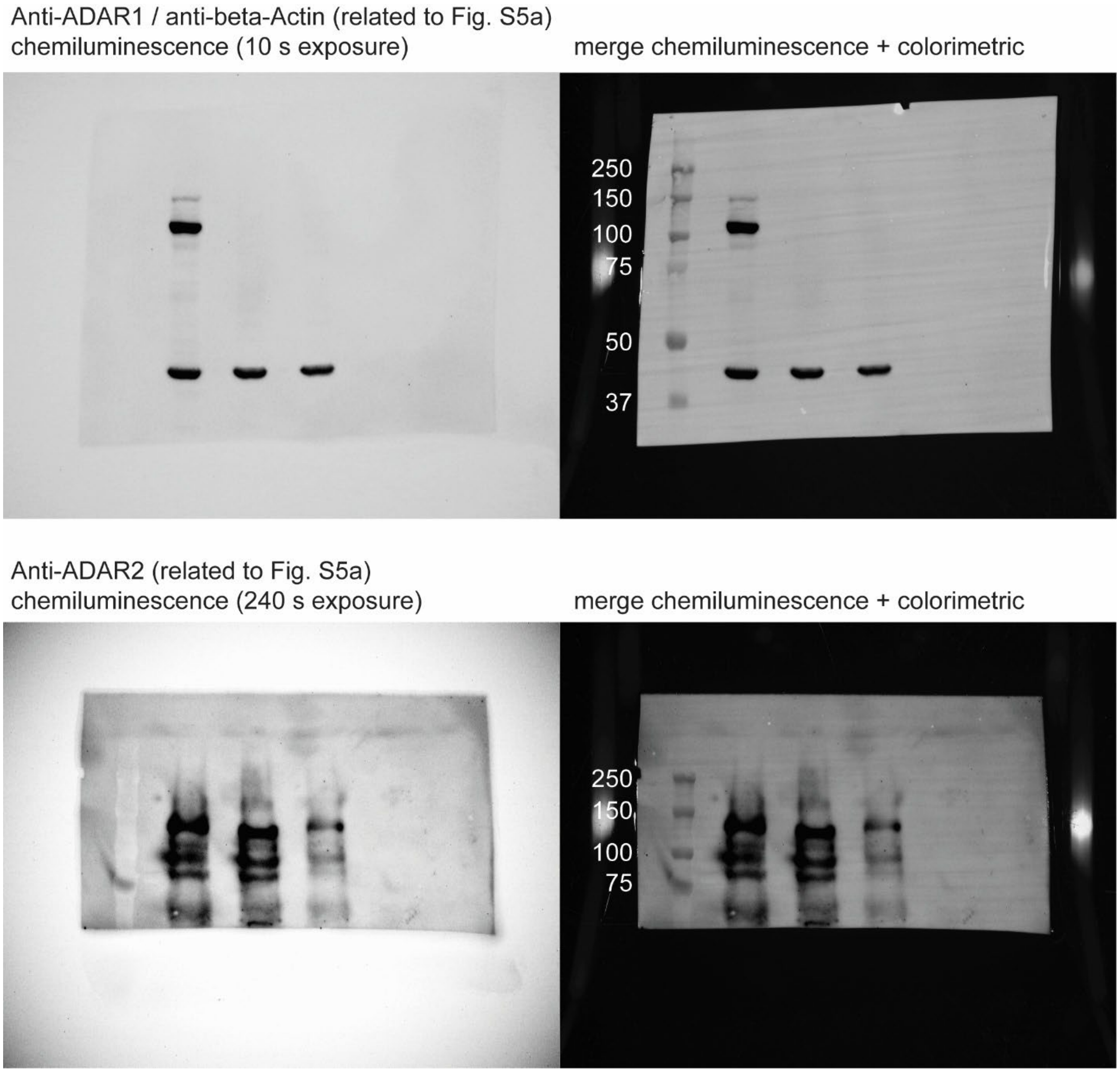

Fig. S15: Uncropped western blot images related to Fig. S5. Top blot was incubated consecutively with anti-ADAR1 / anti-rabbit HRP and anti-beta-Actin-HRP and then imaged on Chemidoc (10 s exposure time). Bottom blot was incubated with anti-ADAR2 / anti-rabbit-HRP and then imaged on Chemidoc (240 s exposure). Each blot is shown as chemiluminescence image only (left) and chemiluminescence + colorimetric images merged to visualize molecular weight marker bands in relation to chemiluminescence signals (right).

Supplementary Table

HeLa WT sample	GEO series (reference)	SRA accession number	total aligned reads (la.stats)	total basecounts (la.stats)
#1	GSE115127 (89)	SRR7239121	72,490,994	6,942,806,051
#2	GSE291675 (86)	SRR32654231	18,745,853	2,665,246,179
#3	GSE291675 (86)	SRR32654230	30,635,357	4,315,890,210
#4	GSE291675 (86)	SRR32654229	38,952,914	5,331,722,344
#5	GSE291675 (86)	SRR32654228	73,635,322	10,361,873,767
HeLa WT		merged	234,460,440	29,617,538,551

HeLa ADAR1-KO sample	GEO series (references)	SRA accession number	total aligned reads (la.stats)	total basecounts (la.stats)
#1	GSE115127 (89)	SRR7239129	79,897,574	7,660,246,503
#2	GSE291675 (86)	SRR32654227	42,409,882	6,057,398,070
#3	GSE291675 (86)	SRR32654226	29,340,497	4,153,759,694
#4	GSE291675 (86)	SRR32654225	163,298,455	23,141,804,958
#5	GSE291675 (86)	SRR32654224	34,006,751	4,706,758,358
HeLa 1KO		merged	348,953,159	45,719,967,583

HeLa ADAR1/2-KO sample	GEO series (references)	SRA accession number	total aligned reads (la.stats)	total basecounts (la.stats)
#1	GSE291675 (86)	SRR32654223	36,221,480	5,142,649,040
#2	GSE291675 (86)	SRR32654222	35,352,916	5,022,601,212
#3	GSE291675 (86)	SRR32654221	49,002,259	6,808,426,923
#4	GSE291675 (86)	SRR32654220	99,290,541	14,016,996,528
HeLa DKO		merged	219,867,196	30,990,673,703

Mouse WT sample	GEO series (references)	SRA accession number	total aligned reads (la.stats)	total basecounts (la.stats)
WT_SRR5223117	GSE94387 (87)	SRR5223117	103,972,742	7,298,014,592
WT_SRR5223118	GSE94387 (87)	SRR5223118	98,685,993	6,895,713,904
WT_SRR5223119	GSE94387 (87)	SRR5223119	100,106,259	7,021,531,826
WT		merged	302,764,994	21,215,260,322

Mouse ADAR1/2-KO sample	GEO series (references)	SRA accession number	total aligned reads (la.stats)	total basecounts (la.stats)
DKO_SRR9203380	GSE132214 (88)	SRR9203380	65,307,831	9,270,462,019
DKO_SRR9203381	GSE132214 (88)	SRR9203381	62,093,975	8,803,815,268
DKO_SRR9203382	GSE132214 (88)	SRR9203382	56,157,381	7,966,100,647
DKO		merged	183,559,187	26,040,377,934

GTEx Brain sample	BioProject number (references)	SRA accession number	total aligned reads (la.stats)	total basecounts (la.stats)
SRR2167030	PRJNA75899 (90)	SRR2167030	48,528,276	11,516,470,808
SRR2167642	PRJNA75899 (90)	SRR2167642	86,255,038	20,432,489,724
SRR2170408	PRJNA75899 (90)	SRR2170408	71,000,698	16,849,467,500
GTEx Brain		merged	205,784,012	48,798,428,032

Ferret PBMC sample	GEO series (references)	SRA accession number	total aligned reads (la.stats)	total basecounts (la.stats)
fPBMC1	GSE291675 (86)	SRR32654219	69,171,850	8,918,063,379
fPBMC2	GSE291675 (86)	SRR32654218	151,744,169	20,317,353,141
fPBMC3	GSE291675 (86)	SRR32654217	66,138,695	8,647,703,525
ferret PBMC		merged	287,054,714	37,883,120,045

Table S1: RNA-seq dataset accession numbers and alignment properties. References are included in the primary reference list.
